# CVD Synthesis of Monodisperse Graphene/Cu Microparticles with High Corrosion Resistance in Cu Etchant

**DOI:** 10.3390/ma11081459

**Published:** 2018-08-17

**Authors:** Shuangyi Li, Baosen Hou, Dan Dai, Shengcheng Shu, Mingliang Wu, Ao Li, Yu Han, Zhi-xiang Zhu, Bao-an Chen, Yi Ding, Qiang Zhang, Qiang Wang, Nan Jiang, Cheng-Te Lin

**Affiliations:** 1 Key Laboratory of Marine Materials and Related Technologies, Zhejiang Key Laboratory of Marine Materials and Protective Technologies, Ningbo Institute of Materials Technology and Engineering (NIMTE), Chinese Academy of Sciences, Ningbo 315201, China; 17855849432@163.com (S.L.); houbaosen@nimte.ac.cn (B.H.); shushengcheng@nimte.ac.cn (S.S.); wumingliang@nimte.ac.cn (M.W.); 15611721208@163.com (A.L.); jiangnan@nimte.ac.cn (N.J.); 2 School of Materials Science and Engineering, Shanghai University, Shanghai 200072, China; 3 College of Physics and Electronic Engineering, Sichuan Normal University, Chengdu 610101, China; 4 University of Chinese Academy of Sciences, 19 A Yuquan Rd., Shijingshan District, Beijing 100049, China; 5 State Key Laboratory of Advanced Transmission Technology, Global Energy Interconnection Research Institute Co., Ltd., Beijing 102209, China; epri313@sina.com (Y.H.); zhuzhixiang003@163.com (Z.Z.); dzcba@163.com (B.C.); dyadin@sina.com (Y.D.); 13601210056@126.com (Q.Z.); 6 State Grid Shanxi Electric Power Company, Taiyuan 030001, China; wq03510351@sina.com

**Keywords:** chemical vapor deposition (CVD) growth, graphene/Cu microparticles, removal spacers, monodisperse, corrosion resistance

## Abstract

Copper powder has broad applications in the powder metallurgy, heat exchanger, and electronic industries due to its intrinsically high electrical and thermal conductivities. However, the ease of formation of surface oxide or patina layer raises difficulty of storage and handling of copper powder, particularly in the case of Cu microparticles. Here, we developed a thermal chemical vapor deposition chemical vapor deposition (CVD) process for large-scale synthesis of graphene coatings on Cu microparticles, which importantly can remain monodisperse without aggregation after graphene growth at high temperature by using removal spacers. Compared to other protective coating methods, the intrinsic electrical and thermal properties of Cu powder would not be degraded by uniform growth of low defect few-layer graphene on each particle surface. As a result, when the anticorrosion performance test was carried out by immersing the samples in Cu etchant, the corrosion rate of graphene/Cu microparticles was significantly improved (ca three times slower) compared to that of pristine Cu powder, also showing a comparable anticorrosion ability to commercial CuZn30 alloy.

## 1. Introduction

Copper powder is an industrial raw material for the fabrication of various electrically and thermally conductive components, such as pantograph contact strips, electrical connectors, and heat sinks [1,2,3]. The widespread applications of copper powder are due to the high electrical (6.0 × 10^7^ S/m) and thermal conductivities (κ: 400 W/mK) after sintering to bulk, and both are only lower than those of silver (6.3 × 10^7^ S/m and 420 W/mK, respectively) among metals [4,5]. However, compared to silver, with the copper surface it is easier to form an oxide or patina layer when exposed to humid air over a long time [6,7], leading to the technical difficulty of storage or transport of copper powder which needs to be hermetically sealed, especially in the cases of micro- and nanoparticles due to their high specific surface area. In order to avoid the surface oxidation and corrosion, some techniques have been extensively employed, such as the formation of corrosion-resistant alloys (Cu-Ni, Cu-Zn, etc.) [8,9], electroplating of the inert metal layers (Cr, Zn, etc.) [10], and cladding with protective organic coatings [11]. However, the use of the above methods also brings some disadvantages, for example, the degradation of thermal conductivity in the alloy system (e.g., ≈272 W/mK in B_0.6_ Cu-Ni alloy) [8], or no longer being compatible with the further production process (like powder metallurgy) when copper powder is coated with a polymer. Therefore, it is strongly required to develop a technique for the improvement of corrosion resistance of copper powder, while maintaining its excellent inherent physical properties.

Graphene, a one-atom-thick carbon layer composed of honeycomb sp^2^-bonded lattice [12], has attracted intense interests in both academia and industry since 2004 [13], due to its ultralow electrical resistivity (theoretically: ≈10^−8^ Ω m), high specific surface area (over 2000 m^2^/g), and chemical inertness [14,15]. Accordingly, diverse applications based on graphene and its derivatives have been proposed and developed over the past ten years. In particular, graphene shows much promise for permeable barrier applications because of its impermeability to standard gases, liquids, and ions in aqueous media [16]. Therefore, graphene as a nanometer-thick barrier has been previously reported, enabling the significant reduction of toxic vapor permeability and an oxidation resistance improvement of metals [17]. Compared to the graphene sheets exfoliated from graphite, the graphene film grown by catalytic chemical vapor deposition (CVD) has much lower defect concentration, showing advantages in preventing the corrosion of metal substrates (Cu, Ni, etc.) in oxidative environments [17,18]. Chen et al. indicated that single-layer CVD graphene provides effective resistance against hydrogen peroxide, thereby protecting the surface of Cu and Cu-Ni alloy [17]. In addition, Parra et al. demonstrated that the Ni surface with graphene coating grown by CVD corrodes five times slower than that covered with mechanically transferred graphene [19]. It suggests that the growth of CVD graphene on copper powder might be able to efficiently protect the surface from oxidation/corrosion, and not degrade the intrinsic electrical/thermal properties. However, considering the melting point of copper (1085 °C), this raises a technical challenge to prevent sintering of copper powder during graphene growth at high temperature (commonly up to 1000 °C) [20,21]. Due to the self-limited growth mechanism, the decrease of CVD temperature would lead to a substantial rise of defect density in the resulting graphene film [22,23]. Alternatively, Lee et al. encapsulated copper particles with a polymer layer as the solid carbon source and then synthesized graphene/copper particles by further annealing [24]. However, the very high ID/IG ratio (≈0.86) in the Raman spectrum indicates the limited quality of the obtained graphene. As a result, the formation of high-quality, low-defect graphene on the surface of copper powder without aggregation is of major importance for the development of potential anticorrosion applications.

In this paper, we report a large-scale synthesis process for preparing Cu microparticles uniformly encapsulated by few-layer graphene. In particular, graphene-coated Cu (Gr/Cu) microparticles remain monodisperse after CVD growth, which was implemented by separation of Cu microparticles with removal graphite spacers to prevent Cu aggregation at high temperature. Compared to other coating techniques, the formation of a thin graphene layer on the surface of each Cu particle almost does not affect its intrinsic electrical and thermal properties. In addition, the corrosion rate of Gr/Cu microparticles was three times reduced compared to the pristine Cu powder and comparable to that of CuZn30 alloy when the samples were immersed in Cu etchant, demonstrating superior anticorrosion ability of Gr/Cu microparticles.

## 2. Experimental

Gr/Cu microparticles were synthesized by thermal CVD in a tube furnace system (BTF-1200C-II-SL, Anhui BEQ Tech., Hefei, China). In order to avoid the coalescence of copper powder at high temperature for graphene growth, graphite particles used as removal spacers (GS) were uniformly mixed with copper powder in advance using a speed mixer (DAC 150.1 FVZ-K, FlackTek Ltd., Hamm, Germany). A 100 g powder mixture with different weight percent ratios was loaded into a CVD chamber, which was heated to 1000 °C at a heating rate of 10 °C/min with an 8 sccm H_2_ flow. When reaching 1000 °C, a gas mixture of H_2_/CH_4_ (8/30 sccm) was introduced for the growth of graphene under 100 Torr for 30 min, followed by cooling down the system with a cooling rate of 20 °C/min. The obtained product was sieved and washed in alcohol 3 times in order to fully collect Gr/Cu microparticles, which were then dried and kept in a desiccator.

The graphene quality was identified by Raman spectrometer (Renishaw plc, Wotton-under-Edge, UK) with 532 nm excitation wavelength of He-Ne laser and X-ray photoelectron spectroscopy (XPS, AXIS ULTR DLD, Kratos Analytical, Hadano, Japan). The morphology, the size distribution, and the crystal structure of the microparticles were determined by a field emission scanning electron microscopy (SEM, QUANTA 250 FEG, FEI, Hillsboro, OR, USA), particle size analyzer (S3500-special, Microtrac Ltd., Montgomeryville, PA, USA), and X-ray diffractometer (XRD, Bruker D8 Advance, Bruker, Karlsruhe, Germany), respectively. The results of corrosion tests were analyzed using inductively coupled plasma-atomic emission spectrometry (ICP, Optima 2100 DV, Perkin Elmer, Waltham, MA, USA) and UV-Vis-NIR Spectrometer (Lambda 950, Perkin Elmer, Waltham, MA, USA).

## 3. Results and Discussion

**Figure 1:**
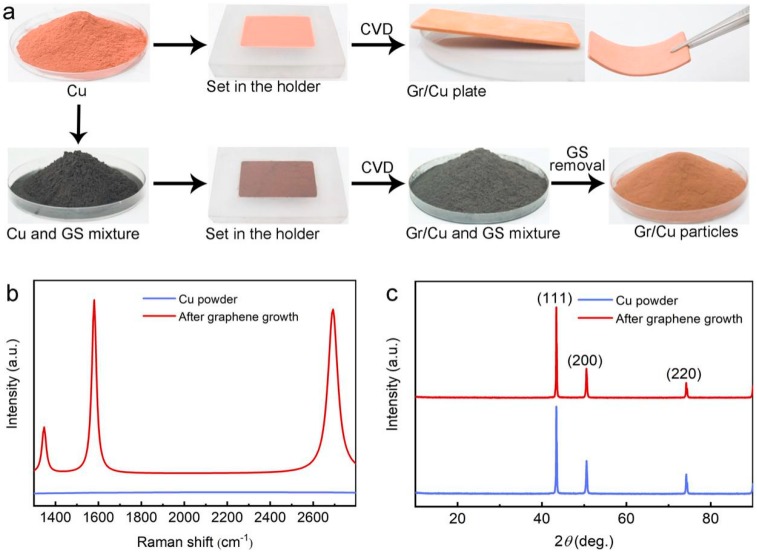
(**a**) Schematic diagram of the synthesis process of monodisperse Gr/Cu microparticles by thermal chemical vapor deposition (CVD); (**b**) Raman and (**c**) X-ray diffraction (XRD) spectra of Cu powder before and after graphene growth.

Figure 1a illustrates our concept of this study to synthesize monodisperse Gr/Cu microparticles by incorporation of GS with Cu powder before the growth of graphene in thermal CVD. Without the use of GS, Cu particles would be sintered to form a porous structure after the CVD process at 1000 °C, because it is close to the melting point of copper (1085 °C). In contrast, the gray mixture of GS and Cu powder remains powdery after graphene growth. After removal of GS by simple mechanical sieving, the color of the obtained sample shows almost no difference to the pristine Cu powder, indicating the success of sintering prevention of the Cu microparticles during CVD using our proposed method.

In order to identify the quality and layer number of graphene formed on the Cu surface, the Raman spectrum of Cu powder after CVD growth was taken and is presented in Figure 1b, which shows typical signals of graphene with peaks corresponding to the D-band (≈1347 cm^−1^), G-band (≈1580 cm^−1^), and 2D-band (≈2691 cm^−1^) [25], while no peak could be observed in the Raman spectra of the pristine sample. The D-band is small with an estimated ID/IG ratio of ≈0.26, which can be attributed to the slight production of defective graphene due to an impurity of pristine Cu (purity: 99.9%) [26]. Moreover, according to the I_2D_/I_G_ ratio (≈0.95) and the full-width at half-maximum (FWHM) of the 2D-band (≈55 cm^−1^), the formation of few-layer graphene on the Cu surface is suggested [27]. Note that no graphene could be synthesized when using the same preparation process but without the introduction of methane (CH4), indicating that GS did not act as carbon source for graphene growth. In the X-ray diffraction (XRD) patterns (Figure 1c), both pristine Cu and Gr/Cu microparticles exhibit typical reflections (111), (200), and (220) of cubic copper (JCPDS No. 04-0836). The absence of a graphite (002) peak at 2θ ≈ 26° confirms the atom-thick nature of the graphene layer deposited on Cu powder [28]. As a result, high-quality, monodisperse Gr/Cu microparticles were successfully prepared using a conventional CVD process with the employment of powder spacers.

**Figure 2:**
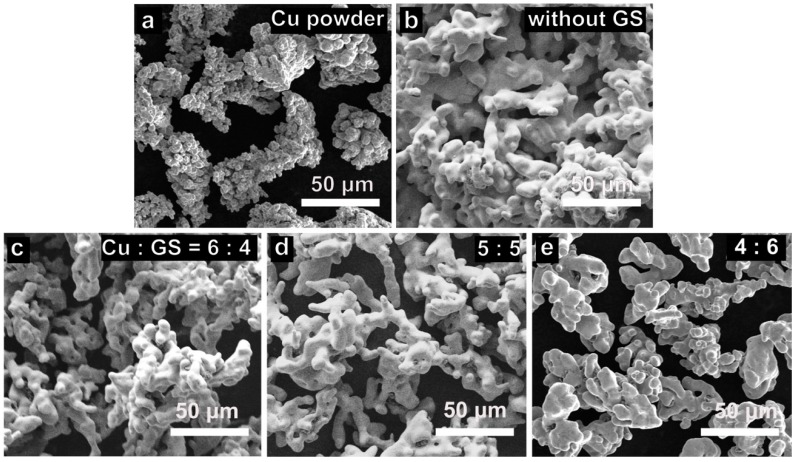
Scanning electron microscopy (SEM) images of (**a**) Cu powder and (**b**) after thermal treatment without the use of removal spacers. Gr/Cu microparticles grown with different mixing ratios of Cu and GS: (**c**) 6:4; (**d**) 5:5; (**e**) 4:6.

The morphological change of Cu powder before and after graphene growth with different mixing ratios of Cu and GS was observed in SEM. In Figure 2a, the pristine Cu powder has a dendritic shape with lateral size in the range of tens of micrometers. The irregular contour of Cu powder is due to its electrolytic production process [29]. Obviously, without pre-mixing with GS, the Gr/Cu powder would be sintered and formed into a porous monolith at high temperature, as exhibited in Figure 2b. In contrast, as the results shown in Figure 2c–e, when GS was added into Cu powder, the synthesized Gr/Cu microparticles remain separated after CVD growth, followed by removal of GS using sieving. In Figure 2c, we found that the Gr/Cu microparticles are still partially agglomerated at the mixing ratio of 4:6 (GS:Cu). As more GS was incorporated, the agglomeration phenomenon was reduced and the size of obtained Gr/Cu microparticles was close to that of pristine powder (see Figure 2d,e). It should be emphasized that the use of GS not only prevents the sintering of Gr/Cu microparticles during CVD, but also creates a flow path to allow complete penetration of methane into the Cu powder, thus guaranteeing the uniform growth of graphene.

**Figure 3:**
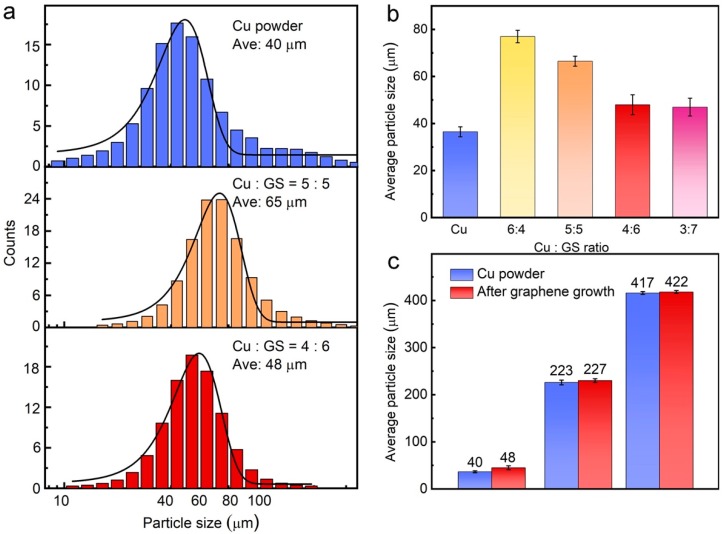
(**a**) The comparison of the size distribution between Cu powder and Gr/Cu microparticles; (**b**) change of the average size of Gr/Cu microparticles prepared with various mixing Cu: GS ratios; (**c**) from Cu powder with three different sizes.

The comparison of the size distribution of Gr/Cu microparticles with various mixing ratios of GS and Cu is presented in Figure 3a. Compared to the pristine Cu powder with a particle size of 40 μm, the average size of Gr/Cu sample is obviously increased when 50% GS is added into the Cu powder before the CVD process. However, when the mixing ratio is 6:4 (GS:Cu), the size distribution of Gr/Cu microparticles shows not much difference (≈48 μm) to that of pristine powder. A systematical study was done and the statistical result is exhibited in Figure 3b, in which we indicate that monodisperse Gr/Cu microparticles could be obtained with no significant size change by optimizing the GS content. Note that the product yield of the Gr/Cu sample would be decreased if the mixing amount of GS was over 60%. Figure 3c manifests that the Gr/Cu microparticles with various particle sizes can be simply prepared using pristine Cu powder with the same size, demonstrating the applicability and effectiveness of the proposed synthesis approach.

**Figure 4:**
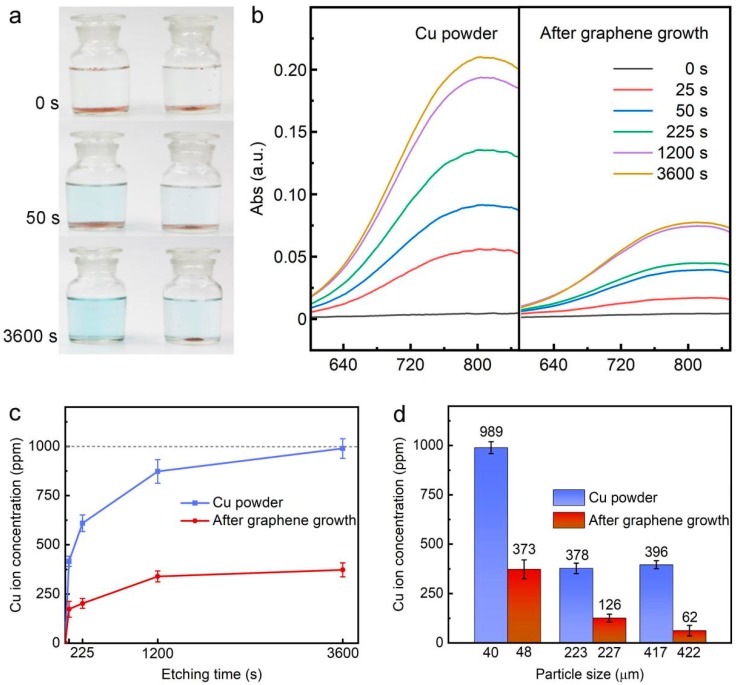
(**a**) Color evolution of Cu powder and Gr/Cu microparticles immersed in Cu etchant (0.5 M ammonium persulfate (APS) solution); (**b**) the comparison of UV-Vis absorption spectra; (**c**) the corresponding dissolved Cu^2+^ concentrations as a function of etching time; (**d**) the superior corrosion resistance property of Gr/Cu microparticles with different sizes.

Graphene has been demonstrated as a diffusion barrier material to effectively prevent the metal bases from oxidation reaction in a corrosive medium [30]. Therefore, a comparison test of anticorrosion property between pristine Cu powder and monodisperse Gr/Cu microparticles was performed by immersing the samples in Cu etchant (APS, ammonium persulfate, ((NH_4_)_2_S_2_O_8_), which is commonly used for graphene transfer after CVD growth [31]. Figure 4a shows the color evolution as a function of etching time when two 0.1 g samples were simultaneously immersed in 0.5 M APS aqueous solution. Obviously, with the increase of immersion time, the etchant solution with pristine Cu powder is consistently bluer than the Gr/Cu microparticles, due to a greater amount of dissolved Cu^2+^ ion in APS solution based on the equation given below [32]:

Cu + S_2_O_8_^2−^ = Cu^2+^ + 2SO_4_^2−^ (1)

After 1 h reaction, we found that the pristine Cu powder was completely dissolved, whereas a lot of Gr/Cu microparticles still remained unaffected, indicating a significant improvement of corrosion resistance of Cu microparticles coated with few-layer graphene. In order to determine the Cu^2+^ ion concentration precisely, a UV-Vis absorption spectrometer was employed because it has an absorption peak at ≈800 nm for the existence of Cu^2+^ ions in APS solution [33]. As presented in Figure 4b, when the immersion time increases, the peak intensity of pristine Cu powder also increases, and is always higher than that of Gr/Cu microparticles.

By comparing to the calibration curve of Cu^2+^ ion concentration versus the absorption intensity at ≈800 nm, the evolution of Cu^2+^ ion concentration as a function of etching time for both samples can be obtained, as shown in Figure 4c. According to the mass of the samples set in APS solution (0.1 g: 100 mL), the maximum concentration of dissolved Cu^2+^ ion is 1000 ppm. In Figure 4c, it can be seen that the etching rate of pristine Cu powder was fast and the sample was completely dissolved after 1 h reaction. In contrast, the weight loss rate of Gr/Cu microparticles was slow and it remained ≈66.8% unreacted even after 1 h etching. The partial removal of Gr/Cu microparticles can be attributed to the permeation of APS solution through the defects of graphene [34]. The anticorrosion performance of Gr/Cu microparticles with different particle sizes was also investigated (Figure 4d). After reaction for 1 h, we found that the etching rate of larger-sized samples is slower due to the smaller specific surface area [35]. However, Gr/Cu microparticles always exhibit superior corrosion resistance property compared to the pristine Cu powder with the same particle size, confirming the barrier effectiveness of graphene coatings.

**Figure 5:**
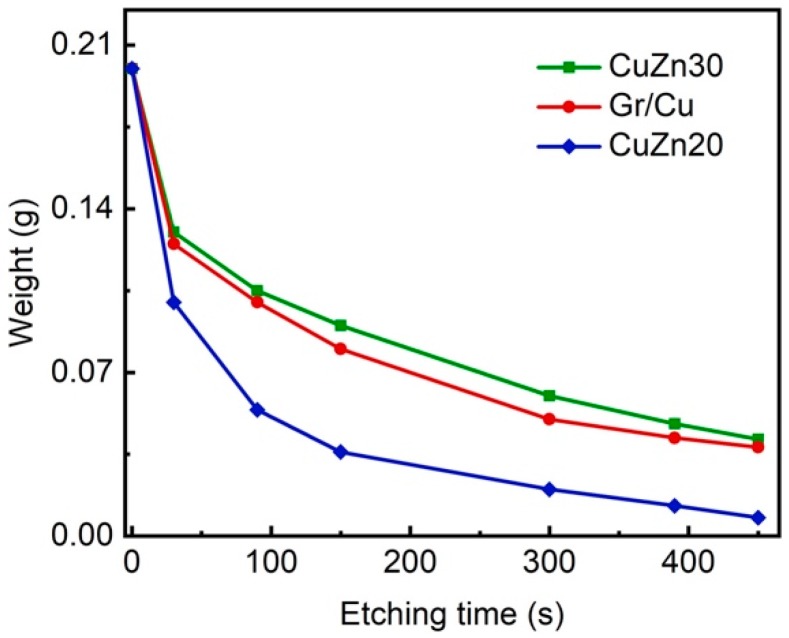
A comparative anticorrosion performance test between Gr/Cu microparticles and Cu-Zn alloy powders.

The formation of Cu-Zn alloys is a common method for improving the corrosion resistance of copper due to dezincification corrosion behavior in the Cu-Zn system [8], however, the drawback is losing the high thermal and electrical conductivities of copper. To further characterize the anticorrosion property of our Gr/Cu microparticles, a comparative study was done by recording the weight loss rate of our samples and Cu-Zn alloy powders (CuZn30 and CuZn20) as a function of etching time in 0.5 M APS solution. As the results in Figure 5 indicate, the rate of weight loss of Gr/Cu microparticles is comparable to that of CuZn30 and higher than that of CuZn20 with the same particle size. This confirms the significant enhancement of corrosion protection efficiency of few-layer graphene coatings on Cu microparticles.

## 4. Conclusions

In summary, we developed a facile CVD process for the synthesis of monodisperse Gr/Cu microparticles in a large quantity using graphite particles as removal spacers, by which the particle size of the obtained products is similar to that of Cu powder and no aggregation is observed, even though the CVD temperature is close to the melting point of copper. The graphene coatings with few-layer thickness on Cu microparticles show superior barrier capability against corrosion in Cu etchant, achieving ca. three times improvement compared to the pristine Cu powder. Moreover, we demonstrated that the anticorrosion performance of Gr/Cu microparticles is comparable to that of CuZn30, suggesting promising applications of our products in powder metallurgy, conductive ink, and electronic materials.
